# High Frequency of Fusion Gene Transcript Resulting From t(10;11)(p12;q23) Translocation in Pediatric Acute Myeloid Leukemia in Poland

**DOI:** 10.3389/fped.2020.00278

**Published:** 2020-07-10

**Authors:** Teofila Ksiazek, Malgorzata Czogala, Przemyslaw Kaczowka, Beata Sadowska, Katarzyna Pawinska-Wasikowska, Mirosław Bik-Multanowski, Barbara Sikorska-Fic, Michał Matysiak, Jolanta Skalska-Sadowska, Jacek Wachowiak, Anna Rodziewicz-Konarska, Alicja Chybicka, Katarzyna Muszynska-Rosłan, Maryna Krawczuk-Rybak, Dominik Grabowski, Jerzy Kowalczyk, Lucyna Maciejka-Kemblowska, Elzbieta Adamkiewicz-Drozynska, Wojciech Mlynarski, Renata Tomaszewska, Tomasz Szczepanski, Joanna Pohorecka, Grazyna Karolczyk, Agnieszka Mizia-Malarz, Katarzyna Mycko, Wanda Badowska, Karolina Zielezinska, Tomasz Urasinski, Irena Karpinska-Derda, Mariola Woszczyk, Małgorzata Ciebiera, Monika Lejman, Szymon Skoczen, Walentyna Balwierz

**Affiliations:** ^1^Department of Medical Genetics, Faculty of Medicine, Jagiellonian University Medical College, Kraków, Poland; ^2^Department of Pediatric Oncology and Hematology, Cytogenetics and Molecular Genetics Laboratory, University Children's Hospital, Kraków, Poland; ^3^Department of Pediatric Oncology and Hematology, Faculty of Medicine, Jagiellonian University Medical College, Kraków, Poland; ^4^University Children's Hospital, Kraków, Poland; ^5^Department of Pediatrics, Hematology and Oncology, Medical University of Warsaw, Warsaw, Poland; ^6^Department of Pediatric Oncology, Hematology and Transplantology, Poznan University of Medical Sciences, Poznań, Poland; ^7^Department of Bone Marrow Transplantation, Pediatric Oncology and Hematology, Medical University of Wroclaw, Wrocław, Poland; ^8^Department of Pediatric Oncology and Hematology, Medical University of Bialystok, Bialystok, Poland; ^9^Department of Pediatric Hematology, Oncology and Transplantology, Medical University of Lublin, Lublin, Poland; ^10^Department of Pediatrics, Hematology and Oncology, University Medical Centre, Gdańsk, Poland; ^11^Department of Pediatrics, Oncology, Hematology and Diabetology, Medical University of Lodz, Łódź, Poland; ^12^Department of Pediatrics Hematology and Oncology, Medical University of Silesia, Zabrze, Poland; ^13^Pediatric Department of Hematology and Oncology, Regional Polyclinic Hospital in Kielce, Kielce, Poland; ^14^Department of Oncology, Hematology and Chemotherapy, John Paul II Upper Silesian Child Heath Centre, The Independent Public Clinical Hospital No. 6 of the Medical University of Silesia in Katowice, Katowice, Poland; ^15^Department of Pediatrics and Hematology and Oncology, Province Children's Hospital, Olsztyn, Poland; ^16^Department of Pediatrics, Hematology and Oncology, Pomeranian Medical University, Szczecin, Poland; ^17^Department of Pediatrics, Hematology and Oncology, City Hospital, Chorzow, Poland; ^18^Department of Pediatric Oncohematology, Clinical Province Hospital of Rzeszów, Rzeszow, Poland; ^19^Department of Genetic Diagnostics, II Department Pediatrics, Medical University of Lublin, Lublin, Poland

**Keywords:** acute myeloid leukemia, 11q23/*KMT2A* rearrangements, *MLL* rearrangements, children, risk stratification, treatment results

## Abstract

11q23/*MLL* rearrangements are frequently detected in pediatric acute myeloid leukemia. The analysis of their clinical significance is difficult because of the multitude of translocation fusion partners and their low frequency. The presence of t(10;11)(p12;q23) translocation was previously identified in pediatric acute myelogenous leukemia (AML). It is considered as the second most common translocation detected in pediatric 11q23/*MLL*-rearranged (present *KMT2A*) AML, after t(9;11)(p22;q23). The presence of the above translocation was previously identified as an unfavorable prognostic factor. Since June 2015, the Polish Pediatric Leukemia/Lymphoma Study Group has applied the therapeutic protocol requiring extensive diagnostics of genetic changes in pediatric AML. Until November 2019, molecular genetic studies were performed in 195 children with diagnosed AML to identify carriers of fusion gene transcripts for 28 most common chromosomal translocations in acute leukemia. The fusion gene transcript for translocation t(10;11)(p12;q23) involving *MLL* gene was detected with unexpectedly high frequency (8.9%) in our research. It was the highest frequency of all detected *MLL* rearrangements, as well as other detected fusion gene transcripts from chromosomal aberrations characteristic for AML. It seems that chromosomal aberration between chromosomes 10 and 11 can be relatively frequent in some populations. Paying attention to this fact and ensuring proper genetic diagnosis seem to be important for appropriate allocation of patients to risk groups of pediatric AML treatment protocols.

## Introduction

Acute myelogenous leukemia (AML) is a heterogeneous group of hematologic malignancies, characterized by unregulated, clonal proliferation of abnormal myeloid progenitor cells. Despite major improvements in outcome over the past decades, it remains a life-threatening malignancy in children. Several constitutive genetic variants and acquired chromosomal abnormalities have been identified as prognostic markers in leukemia (1, 2). In AML in children, genetic diagnostics play an extremely important role in stratifying the risk of treatment failure. Identified genetic markers together with the response to induction treatment are the most important factors that allow selection of optimal therapy for the patient. Among the most relevant factors are chromosomal translocations and gene mutations, which recently have become crucial for risk stratification in pediatric AML (3, 4).

Characteristics for AML genetic changes include structural chromosomal aberrations (translocations, inversions), leading to the formation of gene fusions. The fusion genes present in leukemia cells undergo expression that cause malfunctioning of their protein products (4–6). Common genetic abnormalities in AML with high frequency and well-established favorable prognostic significance include t(8;21)(q22;q22) (*RUNX1-RUNX1T1*), inv(16) (p13;q22) (*CBFB-MYH11*), and t(15;17)(q24;q21) (*PML-RARA*). A basic diagnostic tool for detecting these changes is cytogenetic analysis using the karyotyping technique, supported by fluorescence *in situ* hybridization (FISH). However, nowadays, molecular biology methods are increasingly used to detect transcripts of specific fusion genes in patient's leukemia cells as an important part of the diagnostic process. Molecular techniques have found wide diagnostic application in the detection of not only the best known AML-related fusion genes but also the detection of fusion partners, for example, after the *MLL* (present *KMT2A*) gene rearrangement (7).

A variety of recurrent chromosomal rearrangements involving 11q23/*MLL* gene have been reported in adult and pediatric acute leukemias. So far, more than 135 different fusion partners of *MLL* gene have been identified in acute leukemias (8). Most common for pediatric AML are translocations t(9;11)(p21.3;q23.3) (*MLL-MLLT3*), t(10;11)(p12;q23.3) (*MLL-MLLT10*), t(11;19)(q23.3;p13.1) (*MLL-ELL*), and t(6;11)(q27;q23.3) (*MLL-AFDN*) (7–9). The prognostic impact of *MLL* rearrangement depends on detected fusion partner but is not always clearly defined. However, the presence of translocation t(10;11)(p12;q23) is usually associated with a poor prognosis (3, 7, 9).

## Materials and Methods

Since June 2015, the Polish Pediatric Leukemia/Lymphoma Study Group has applied the therapeutic protocol for pediatric AML requiring extensive diagnostics of genetic changes in leukemic cells. For all patients, conventional and molecular cytogenetics analyses were recommended—classical karyotype and FISH analyses for the most common translocation in AML: t(8; 21)(q22; q22)/*RUNX1-RUNX1T1*, inv(16)(p13; q22)/*CBFB-MYH11*, t(15;17)(q24; q21)/*PML-RARA*, and *MLL* rearrangements by break-apart FISH probes. Molecular analyses were also performed to screen for further, less common transcripts of fusion genes founded in leukemia, as well as mutation in *WT1, FLT3, NPM1, CEBPA*, and *GATA1* genes. Until November 2019, molecular genetic studies were performed in 195 children with *de novo* diagnosed AML. This represents nearly all pediatric patients with AML in Poland during this period.

One of the performed molecular analyses was genotyping for carriers of fusion gene transcripts for 28 most common chromosomal translocations with prognostic significance in leukemia by a CE-marked (approved for use in Europe) *in vitro* diagnostic test based on multiplex reverse transcription–polymerase chain reaction (RT-PCR) screening assay, HemaVision-28N RT-PCR (DNA Diagnostic A/S, Risskov, Denmark). The detailed information about 28 leukemia-causing translocations detected by HemaVision-28N is presented in Supplement Table 1. Diagnostic tests were conducted on mRNA samples isolated from mononuclear cells from bone marrow collected before the treatment. For this purpose, bone marrow samples were centrifuged in a density gradient (Histopaque-1077; Sigma-Aldrich, St. Louis, MO, USA), and the obtained bone marrow mononuclear cells were lysed in TRI Reagent Solution (Thermo Fisher Scientific Baltics, Vilnius, Lithuania). The isolation of total RNA was performed according to the manufacturer's instructions. Next, 2 μg of non-degraded RNA was used as a template for synthesis of cDNA in RT reaction (SuperScript™ II Reverse Transcriptase; Thermo Fisher Scientific). HemaVision RT-PCR assay used cDNA as a template for multiplex PCR amplification reactions, followed by nested PCR reactions. All PCR reactions were performed with primer mixes from the HemaVision HV01-28N kit and Multiplex PCR Master Mix (Eur_x_, Gdansk, Poland). The final PCR products were analyzed by agarose gel electrophoresis.

All children were treated according to the same therapeutic protocol—AML BFM 2012/2019. The genetic tests and individual persons' data used in publication were obtained and processed according to written informed consent obtained from guardians of all patients in accordance with the Declaration of Helsinki. The study was approved by the Ethical Committee of the Jagiellonian University in Krakow (KBET 122.6120.17.2015, dated January 29, 2015). The data that support the findings of the study are available on request from the corresponding author.

## Results

**Figure 1:**
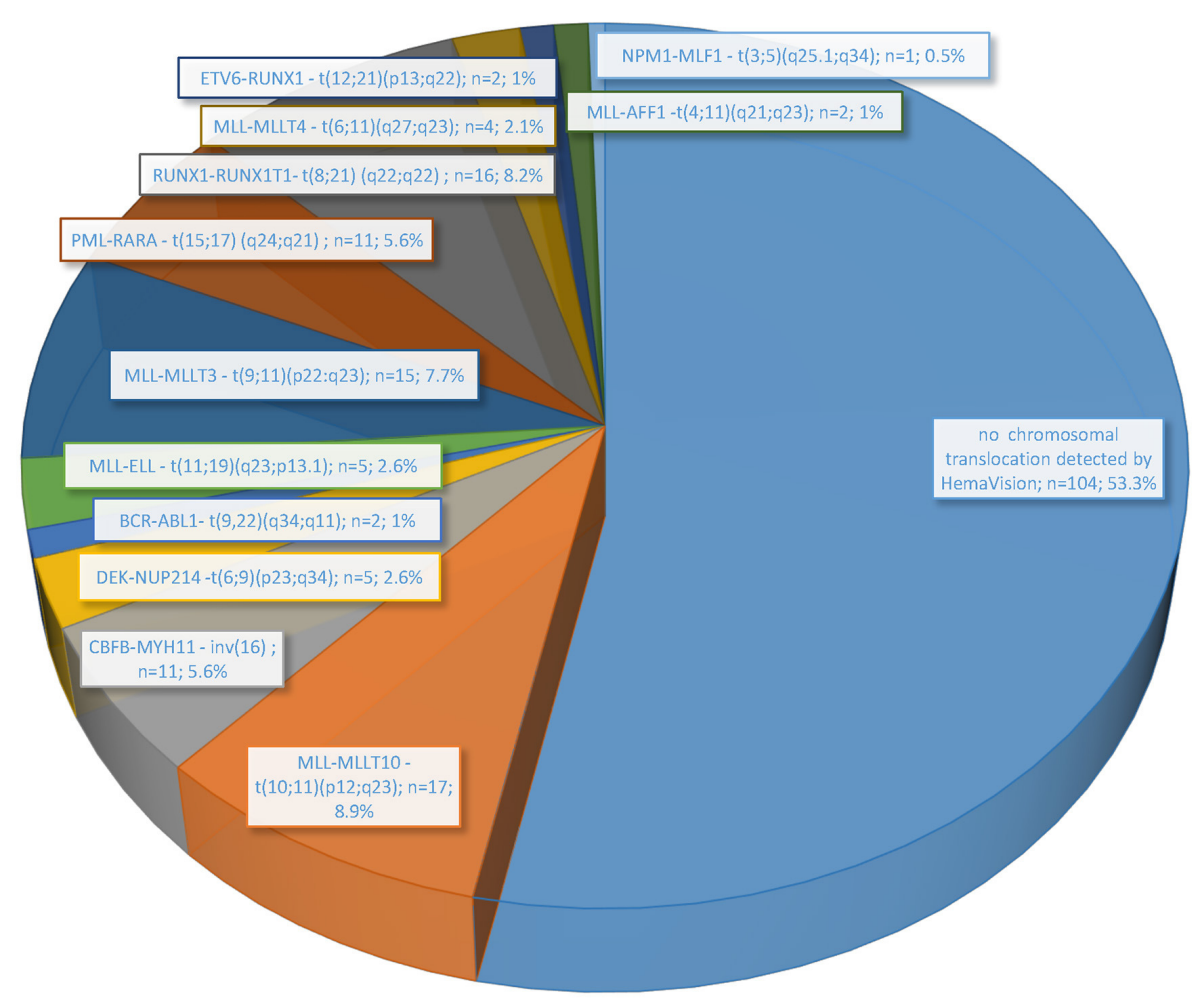
Frequency of chromosomal translocations associated with leukemia detected by molecular methods (HemaVision RT-PCR; DNA Diagnostic A/S) in 195 pediatric AML patients treated in Poland from June 2015 to November 2019.

In the performed molecular genetic studies, the presence of fusion genes transcripts was revealed in 46.7% (91/195 patients) (Figure 1). The fusion gene transcript for translocation t(10;11)(p12;q23) (*MLL-MLLT10*) was detected in 8.9% (17/195) of cases with surprisingly the highest frequency among all other marked chromosomal abnormalities determined in HemaVision-28N. Slightly lower frequency was observed for fusion transcripts generated by chromosomal translocation t(8;21) (q22;q22) (*RUNX1-RUNX1T1*) (8.2%; 16 patients) and t(9;11)(p22:q23) (*MLL-MLLT3*) (7.7%; 15 patients). Furthermore, other AML-characteristic transcripts related to 11q23/*MLL* rearrangements, such as t(11;19)(q23;p13.1), t(6;11)(q27;q23), and t(4;11)(q21;q23), were also found. Together, five different fusion gene transcripts for 11q23/*MLL* rearrangements were detected in 43 patients (22.1%).

For 14 of 17 children with molecularly confirmed of *MLL-MLLT10* fusion transcript, the results of karyotype and FISH analysis were obtained, and a summary is shown in Table 1. In some cases, the karyotype result indicated other position of chromosome breaks than expected due to molecular result, especially in the case of chromosome 10 (p11, p13, p?). Pediatric karyotypes always pose a great diagnostic challenge due to achieved resolution and G-band quality. Obtained cytogenetic results (karyotype, FISH) were performed in local laboratories, and molecular verification was carried out in a reference laboratory. No correction of chromosome breakpoints assessed in the classical karyotype after molecular analysis results was performed.

**Table 1:** Clinical and genetic characteristics of 17 children with diagnosed AML and confirmed t(10;11)(p12;q23) fusion gene transcript in the molecular genetic study.

**Case no**.	**Sex**	**Age at diagnosis**	**FAB**	**% Leukemic blasts in BM**	**Treatment results**	Karyotype*	**FISH analysis of *MLL* rearrangements**
1	F	<6 months old	M5	77	CR, HSCT	47,XX,der(1)t(1;10;11)(q32;p13;q23)del(1)(p22.1)?add(1)(p22.1) dup(1)(q21q32,der(10)t(1;10;11)(q32;p13;q23) ins(10;11)(p13;q23q23),der(11)t(1;10;11)(q32;p13;q23),+19	Positive
2	F	6 months old	M5	66.6	Death due to pulmonary embolism in early relapse treatment	48,XX,+6,der(10)(p?),+19	No data
3	M	16 years old	M5	78.2	CR, HSCT	46,XY,der(3)t(3;19),t(10;11)(p13;q23),r1(19)/47,idem,+r2	No data
4	M	17 years old	M5	85	Death due to pulmonary leukostasis, acute liver failure and cardiopulmonary failure before achieving remission	46,XY	Positive ish der(9)t(9;10)(p21;p12)(wcp10+),der(10)t(10;11)(p11;q23)(wcp10+,wcp11+),der(11)t(11;10;9)(p11pter-q23::10p11 -10p12::9p21-9pter)(wcp11+,wcp10+)[20]
5	M	1 year old	M5	77	Death in CR in course of cytomegalovirus infection after HSCT	46,XY,t(10;11)(p11.2q23)	Positive
6	F	1 year old	M5	76.5	CR, HSCT	50,XX,+8, +8, t(10;11)(p12;q23?),+20, +21/54-57,XX,+4,+5,+6,+8+8,t(10;11)(p12;q23"),+12,+16,+18,+19,+20,+21;+21	Positive
7	F	10 years old	M5	41	CR, HSCT	54-56, X, t(X;10;11)(q22;p13;q23),+5,+6,+8,+8,+10, del(10)(p13),+14,+18,+19,+21,+21 [cp19]	Positive
8	M	<1 year old	Myeloid Sarcoma	6.4	CR, HSCT	No cell division	Positive
9	F	3$\frac{1}{2}$ years old	M5	97	CR, HSCT	46,XX,t(10;11)(p12;q23)	Positive
10	F	5 years old	M0	No data	CR, HSCT	No data	No data
11	F	7 years old	M5	98.2	CR, HSCT	No data	No data
12	M	6 years old	M5	69	Early relapse	No data	No data
13	M	12 years old	M5	88	CR, HSCT	46,XY,t(10;11)(p12;q13),inv(11)(q13q23)	Positive
14	F	7 years old	No data	No data	No data	46,XX,t(10;11)(p12;q23),del(12)(p12),del(16)(q12)/46,XX	Positive
15	M	1$\frac{1}{2}$ years old	No data	No data	No data	46,XY,t(10;11)(p12;q23),t(12;20)(p11.2;p13) /46,XY,t(12;20)(p11.2;p13)	Positive
16	F	<6 months old	M5	81.6	CR, HSCT	46,XX	Positive ish der(10)ins(10;11)(p12;q13q23)(5′KMT2A+)(2) nuc ish(5′KMT2Ax3,3′KMT2Ax2)(5′KMT2A con 3′KMT2Ax2)[184/230]
17	F	<1 year old	M5	76.4	Death due to sepsis and ischemic stroke before achieving remission	46,XX,t(7;10)(q11.2;p11.2)/46,XX	No data

*M, male; F, female; BM, bone marrow; CR, complete remission; HSCT, allogenic hematopoietic stem cell transplantation*.

* *In some cases, the karyotype result indicated other position of chromosome breaks than expected due to molecular result. Obtained cytogenetic results (karyotype, FISH) were performed in local laboratories, and molecular verification was carried out in a reference laboratory. No correction of chromosome breakpoints assessed in the classical karyotype after molecular analysis results was performed*.

In four of those 14 patients, only rearrangements in chromosomes 10 and 11 were observed (cases 5, 9, 8, and 16, for whom rearrangements of *MLL* gene were confirmed only by FISH). For the next seven cases, additional chromosomal abnormalities or complex karyotype was detected (cases 1, 3, 6, 7, 13, 14, and 15; the results in detail in Table 1). For patient 4, the karyotyping result was normal, but in a metaphase FISH, the variant translocation with chromosomes 11, 10, and 9 was confirmed (cryptic rearrangement). Moreover, variant translocations were also observed in two other cases—patient 1: t(1;10;11) and patient 7: p t(X;10:11). It should be emphasized that in all these cases the fusion gene transcript for *MLL-MLLT10* was detected in molecular studies. For the last two children (cases 2 and 17), the presence of translocation t(10;11)(p12;q23) was not simply confirmed; however, abnormalities in chromosome 10 were indicated in both cases. Unfortunately, no data from FISH analysis were available for them. In the case of low G-band quality and resolution in the karyotype analysis for children with leukemia, such results do not exclude rearrangement of chromosomes 10 and 11 for patient 2 or cryptic variant translocation of chromosomes 7, 10, and 11 for patient 17.

The clinical characteristics of 17 presented children with diagnosed AML and molecular confirmation of *MLL-MLLT10* fusion gene transcript are presented in Table 1. All of the children were treated according to the AML-BFM 2012/2019−10 girls and 7 boys with median age of 3.6 years (range, 4 months to 16.2 years). In four of them, hyperleukocytosis (91–245 × 10^9^/L) was observed at diagnosis. Ten patients achieved complete remission (CR) and underwent allogenic hematopoietic stem cell transplantation (HSCT). Two patients died early before achieving remission—the first because of sepsis and ischemic stroke 1.4 months after diagnosis and the second one due to pulmonary leukostasis, acute liver failure, and cardiopulmonary failure 13 days after final diagnosis of AML. Two further patients had an early relapse; one of them died because of pulmonary embolism. One patient died in CR in course of cytomegalovirus infection 2.6 months after HSCT. Median observation time was 11.5 months (range, 0.4–49.7 months). For two patients, no follow–up data were available.

## Discussion

The presence of t(10;11)(p12;q23) translocation was previously identified in pediatric AML and considered as an unfavorable prognostic factor, but it was observed rarely (1, 3, 8–11). This rearrangement is described in the latest large cohort studies as the second most common translocation detected in pediatric 11q23/*MLL*-rearranged AML, after t(9;11)(p22;q23) (8). Its occurrence is being estimated at 3% (3). Surprisingly, in our research, the fusion gene transcript resulting from t(10;11)(p12;q23) frequency (8.9%) was not only the highest in the case of all detected *MLL* rearrangements but also in relation to other common chromosomal translocation in AML, among others t(8;21)(q22;q22) (*RUNX1-RUNX1T1*)-−8.2% (16/195 patients). Similar results were presented by Stasevich et al. (12), who confirmed the presence of the t(10;11)(p12;q23) at a very high level in children treated for AML in 1 year at Minsk Oncology Center. In the mentioned article, five children with of t(10;11)(p12;q23) translocation were being described among 18 pediatric patients. Worth noting, all of five children do not have the simple reciprocal translocation; in all cases, MLL-MLLT10 fusion was created by several rearrangements. Also, in our research, in 3 of 13 children with karyotype result, variant translocation of 11q23/*MLL* was revealed [t(1;10;11), t(11;10;9), t(X;10;11)]. Therefore, it seems that in some cases the ambiguity and complexity of changes detected by cytogenetic analysis do not always allow for a correct and rapid assessment of the presence of rearrangement of chromosomes 10 and 11 in leukemic blasts. Hence, the molecular genetic studies seem to be a more appropriate technique to unequivocal assessment of the presence of *MLL-MLLT10* fusion transcript, especially in a case of possible missing of this chromosomal aberration during karyotyping (two children with normal karyotype reported) or when no karyotyping result is obtained.

In the presented group of 17 patients with positive molecular results for *MLL-MLLT10*, in three patients (patients 10–12 in Table 1), there is no cytogenetic confirmation of the results because of the lack of access to archival data. For the next two patients (patients 2 and 17), only the karyotype analysis was performed, with the results not indicating the presence of *MLL* rearrangement. For this reason, no FISH analysis was performed in these cases. However, in both these cases, chromosome breakage in 10p was observed [48,XX,+6,der(10)(p?),+19 and 46,XX,t(7;10)(q11.2;p11.2)/46,XX]. In the context of a positive molecular result for the *MLL-MLLT10* fusion gene transcript, the presence of more complex chromosomal rearrangements, including chromosome 11 rearrangement, may be possible in these patients (cryptic or variant translocation). This also indicates the need for obligatory FISH for *MLL* rearrangements in all AML patients.

Therefore, because of the large amount of molecular diagnostic tests performed with the HemaVision-28N RT-PCR IVD assay (for 195 children diagnosed with AML) and cytogenetic confirmation of the obtained result in the 12 presented cases, also the molecular results for this group of five patients without direct cytogenetic confirmation were considered reliable and included in the analysis.

According to the recommendations of an international expert panel, the patients with translocation t(10;11)(p12;q23) should be allocated to the high-risk group of pediatric AML treatment protocols (3). The MLL gene encodes a histone methyltransferase that positively regulates homeobox (HOX) gene expression (13). The presence of MLL-MLLT10 fusion protein observed in patients with t(10;11)(p12;q23) in leukemic blasts affects the histone methylation ability and dysregulation of HOXA and MEIS1 expression, finally leading to MLL rearrangement-dependent leukemia (14). It cannot be excluded that the presence of the *MLL-MLLT10* fusion transcript detected by molecular methods in the case of variant chromosomal rearrangements or complex karyotype does not have an equally significant negative effect on cell function. However, further research is required in this matter. To sum up, it seems that fusion gene transcript resulting from t(10;11)(p12;q23) can be relatively frequent in some populations, or its frequency may be underestimated because of the difficulty of its cytogenetic assessment. Paying attention to this fact and ensuring proper genetic diagnosis seem to be important for appropriate allocation of patients to risk groups of pediatric AML treatment protocols.

## Data Availability Statement

The datasets generated for this study are available on request to the corresponding author.

## Ethics Statement

The studies involving human participants were reviewed and approved by Ethical Committee of the Jagiellonian University in Krakow. Written informed consent to participate in this study was provided by the participants' legal guardian/next of kin.

## Author Contributions

TK, MCz, and WBal: study concept and design, acquisition of data, analysis and interpretation of data, drafting of the manuscript, and critical revision of the manuscript for important intellectual content. PK, BS, and ML: analysis and interpretation of data and critical revision of the manuscript for important intellectual content. KP-W: analysis and interpretation of data assistance, acquisition and accumulation of data, and critical revision of the manuscript for important intellectual content. MB-M and SS: critical revision of the manuscript for important intellectual content. BS-F, MM, JS-S, JW, AR-K, AC, KM-R, MK-R, DG, JK, LM-K, EA-D, WM, RT, TS, JP, GK, AM-M, KM, WBad, KZ, TU, IK-D, MW, and MCi: acquisition of data. All authors contributed to the article and approved the submitted version.

## Conflict of Interest

The authors declare that the research was conducted in the absence of any commercial or financial relationships that could be construed as a potential conflict of interest.
