# Supplementary material for: High Frequency of Fusion Gene Transcript Resulting From t(10;11)(p12;q23) Translocation in Pediatric Acute Myeloid Leukemia in Poland
Source: Front Pediatr. 2020 Jul 10;8:278. doi: 10.3389/fped.2020.00278 (PMC7366384; doi:10.3389/fped.2020.00278)
Supplement: Supplementary file 1 [file Table_1.pdf]

Supplement Table A. 28 leukemia causing chromosomal translocations detected by a HemaVision-28N CE-marked in vitro diagnostic test (DNA Diagnostic A/S, Denmark).

**Chromosome aberration**	***Fusion gene***
del1(p32)	*STIL-TAL1*
t(1;11)(p32;q23)	*MLL-EPS15*
t(1;11)(q21;q23)	*MLL-MLLT11*
t(1;19)(q23;p13)	*TCF3-PBX1*
t(3;5)(q25;q34)	*NPM1-MLF1*
t(3;21)(q26;q22)	*RUNX1-MDS1/EVI1*
t(4;11)(q21;q23)	*MLL-AFF1*
t(5;12)(q33;p13)	*ETV6-PDGFRB*
t(5;17)(q35;q21)	*NPM1-RARA*
t(6;9)(p23;q34)	*DEK-NUP214*
t(6;11)(q27;q23)	*MLL-MLLT4*
t(8;21)(q22;q22)	*RUNX1-RUNX1T1*
t(9;9)(q34;q34)	*SET-NUP214*
t(9;11)(p22;q23)	*MLL-MLLT3*
t(9;12)(q34;p13)	*ETV6-ABL1*
t(9,22)(q34;q11)	*BCR-ABL1*
t(10;11)(p12;q23)	*MLL-MLLT10*
t(11;17)(q23;q21)	*MLL-MLLT6*
t(11;17)(q23;q21)	*ZBTB16-RARA*
t(11;19)(q23;p13.1)	*MLL-ELL*
t(11;19)(q23;p13.3)	*MLL-MLLT1*
t(12;21)(p13;q22)	*ETV6-RUNX1*
t(12;22)(p13;q11**)**	*ETV6-MN1*
t(15;17)(q24;q21)	*PML-RARA*
inv(16)(p13;q22)	*CBFB-MYH11*
t(16;21)(p11;q22)	*FUS-ERG*
t(17;19)(q22;p13)	*TCF3-HLF*
t(X;11)(q13;q23***))***	*MLL-FOXO4*
